# Pre‐ and post‐diagnostic meat intake in relation to risk of recurrence and mortality among individuals with stage I–III colorectal cancer

**DOI:** 10.1002/ijc.70113

**Published:** 2025-09-18

**Authors:** Anne‐Sophie van Lanen, Dieuwertje E. Kok, Evertine Wesselink, Jeroen W. G. Derksen, Anne M. May, Karel C. Smit, Miriam Koopman, Johannes H. W. de Wilt, Ellen Kampman, Fränzel J. B. van Duijnhoven, the COLON and PLCRC studies, Hester van Cruijsen, Jan Willem T. Dekker, Henk K. van Halteren, Johan J.B. Janssen, Maartje Los, Anandi H. W. Schiphorst, Dirkje W. Sommeijer, Dirk J. A. Sonneveld, Mark P. S. Sie, Maarten Vermaas

**Affiliations:** ^1^ Division of Human Nutrition and Health Wageningen University & Research Wageningen The Netherlands; ^2^ Julius Center for Health Sciences and Primary Care University Medical Center Utrecht, Utrecht University Utrecht The Netherlands; ^3^ Department of Medical Oncology University Medical Center Utrecht, Utrecht University Utrecht The Netherlands; ^4^ Department of Surgery Radboud University Medical Center Nijmegen The Netherlands

**Keywords:** all‐cause mortality, colorectal cancer recurrence, processed meat, unprocessed poultry, unprocessed red meat

## Abstract

Processed meat and unprocessed red meat intakes are associated with increased colorectal cancer (CRC) risk, but evidence on associations with mortality after a CRC diagnosis is inconsistent. To date, no studies examined associations between unprocessed poultry intake and mortality, or assessed cancer recurrence risk as a separate outcome measure. We included data from 2484 individuals, who were newly diagnosed with stage I–III CRC, participating in 2 prospective cohort studies. Dietary intake was assessed at diagnosis and 6 months after diagnosis. Multivariable Cox proportional hazards regression models and restricted cubic splines were used to examine associations between pre‐ and post‐diagnostic meat intake and risk of recurrence and all‐cause mortality. We performed subgroup analyses by sex, disease stage and primary tumour location. During a median follow‐up time of 5.0 years for recurrence analyses and 6.4 years for mortality analyses, 336 recurrences and 409 deaths occurred. Pre‐ and post‐diagnostic processed meat and unprocessed red meat intakes were not associated with risk of recurrence nor all‐cause mortality. At both timepoints, a higher unprocessed poultry intake was non‐linearly associated with a decreased mortality risk, with the lowest risk observed at 20 g/day (hazard ratio: 0.63, 95% confidence interval: 0.47–0.85), compared to 0 g/day. Results were not substantially different by sex, disease stage and primary tumour location. To conclude, a higher pre‐ and post‐diagnostic intake of unprocessed poultry, but not processed meat and unprocessed red meat, was associated with a decreased all‐cause mortality risk in individuals with stage I–III CRC. Future studies in independent study populations should confirm these findings.

## Graphical abstract

What's new?

Processed meat and unprocessed red meat intakes are associated with increased colorectal cancer risk. However, evidence on their associations with mortality after colorectal cancer diagnosis is inconsistent. This study examined associations between three meat types, including unprocessed poultry, and the risk of recurrence and all‐cause mortality in stage I–III colorectal cancer. During a median follow‐up time of 5.0 years for recurrence and 6.4 years for mortality analyses, pre‐ and post‐diagnostic processed meat and unprocessed red meat intakes were not associated with risk of recurrence or all‐cause mortality. A higher intake of unprocessed poultry was associated with a decreased all‐cause mortality risk.

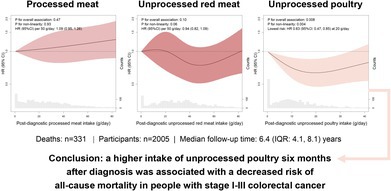

**Abbreviations**
BMI: body mass index
CRC: colorectal cancer
HR: hazard ratio
IQR: interquartile range
SQUASH: Short QUestionnaire to ASsess Health‐enhancing physical activity
95% CI: 95% confidence interval

## 1 INTRODUCTION

There is strong evidence that processed meat and red meat intakes are associated with an increased risk of colorectal cancer (CRC).^1^ As a result, the World Cancer Research Fund (WCRF) and the American Institute for Cancer Research have incorporated the advice to consume as little processed meat as possible and to limit red meat intake to 500 g/week in cancer prevention recommendations.^1^ However, unlike the established associations with CRC risk, it is unclear whether pre‐ and post‐diagnostic intakes of processed meat and red meat influence the risk of recurrence and mortality following a CRC diagnosis.

In a recent pooled analysis of 10 studies, including 7627 participants with stage I–III CRC, a higher pre‐diagnostic intake of processed meat was associated with an increased risk of all‐cause mortality when comparing participants with an intake above versus below the study‐specific median intake (hazard ratio [HR]: 1.12, 95% confidence Interval [CI]: 1.01, 1.25).^2^ However, when comparing upper versus lower quartiles of intake, neither processed meat nor red meat was statistically significantly associated with the risk of mortality. Three prospective cohort studies on post‐diagnostic processed meat and unprocessed red meat intakes in relation to survival have yielded heterogeneous results.^3^, ^4^, ^5^ It must be noted that the study populations were very different regarding sex (female or male only), disease stage at diagnosis, and primary tumour location, and also the timing of post‐diagnostic dietary assessment differed substantially between studies.

Risk of recurrence may depend on sex, stage at diagnosis and primary tumour location^6^, ^7^ and risk factors for recurrence may be different for individuals with colon compared to those with rectal cancer.^7^ It could be that associations between meat intake and risk of recurrence or all‐cause mortality are restricted to specific subgroups of individuals. In the previously mentioned pooled analysis,^2^ no statistically significant interactions were observed for red meat and processed meat in combination with sex or tumour location in relation to CRC‐specific or all‐cause mortality. However, this study did not study unprocessed poultry intake as exposure, risk of recurrence as outcome, nor include disease stage in subgroup analyses. Also, one cohort study including 529 adults newly diagnosed with stage I–IV CRC did observe that a processed meat dietary pattern was only associated with worse disease‐free and overall survival in female participants and in those with colon cancer.^8^ Therefore, the current study includes subgroup analyses based on sex, disease stage and primary tumour location.

One of the suggested mechanisms underlying the associations between red meat intake and CRC risk is the endogenous production of carcinogenic N‐nitroso compounds stimulated by heme iron intake.^1^, ^9^ Also, dietary heme has been described to induce cytotoxicity in the colonic lumen in rodents.^10^ This cytotoxic colonic content can damage colonic epithelial cells and induce compensatory hyperproliferation,^10^ possibly via gut microbiota that reduce the mucus barrier function.^11^ Heme iron content in meat products can vary with cooking method and doneness level,^12^ but poultry and pork generally contain lower amounts of heme iron than beef^12^, ^13^ and lamb.^13^ Although evidence is limited,^1^ higher poultry intake has been associated with a reduced risk of CRC.^14^ To our knowledge, no study thus far has investigated associations between pre‐ or post‐diagnostic intake of unprocessed poultry in relation to the risk of recurrence or mortality.

The majority of studies investigating meat intake in relation to CRC prognosis have studied pre‐diagnostic intakes, and none have investigated its relation to risk of CRC recurrence directly as an independent outcome measure. As the majority of cancer recurrences occur within 3 years after diagnosis,^7^ we hypothesise that the period before and shortly after diagnosis is most relevant when studying associations between meat consumption and risk of recurrence.

Therefore, the current study assessed processed meat, unprocessed red meat and unprocessed poultry intake before and 6 months after CRC diagnosis in relation to the risk of recurrence and all‐cause mortality in people with stage I–III CRC. Additionally, we studied these associations in subgroups of sex, primary tumour location and disease stage.

## 2 METHODS

### 2.1 Study population

**FIGURE 1:**
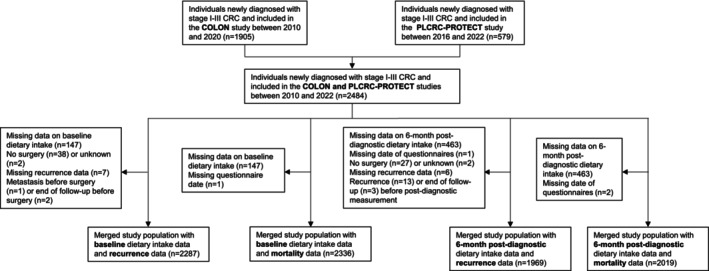
Flow chart.

The initial study population consisted of 2,484 adults who were newly diagnosed with stage I–III CRC, from two Dutch prospective cohort studies: the COLON study (*n* = 1905, ClinicalTrials.gov Identifier NCT03191110)^15^ and the PLCRC‐PROTECT study (*n* = 579, ClinicalTrials.gov Identifier NCT02070146) (Figure 1).^16^, ^17^ Details of the COLON study and overall PLCRC study have been described previously.^15^, ^16^, ^17^ Briefly, participants of the COLON study were recruited after a CRC diagnosis from 11 hospitals in the Netherlands between August 2010 and February 2020. Recruitment for the PLCRC‐PROTECT study was ongoing at the time of data analysis. Participants who were recruited from 21 hospitals between February 2016 and June 2022 were included in the current study population.

### 2.2 Assessment of exposure

In both studies, participants filled out an identical self‐administered semi‐quantitative food frequency questionnaire (FFQ) of 204 items at diagnosis and 6 months after diagnosis,^18^, ^19^ reflecting on dietary intake in the month prior to diagnosis and the month prior to filling out the post‐diagnostic questionnaire, respectively. Dietary intake of processed meat, unprocessed red meat and unprocessed poultry was calculated in grams per day, based on frequency of consumption and portion size, using the online Dutch Food Composition Table (version 2011/3.0).^20^ Processed meat included all red meat and poultry that has been processed, including lunch meats, ham, sausages and salami. Unprocessed red meat included all fresh mammalian muscle meat (e.g., pork, beef and lamb), including fresh minced meat. Unprocessed poultry included fresh chicken and turkey.

### 2.3 Assessment of covariates

At diagnosis and 6 months after diagnosis, participants from both cohorts also filled out a general questionnaire on demographics, anthropometrics, cancer family history and lifestyle habits, including questions about age (years), sex (female/male), education (low/medium/high), body weight (kg), height (cm) and smoking status (current/former/never). Physical activity was assessed using the Short QUestionnaire to ASsess Health‐enhancing physical activity (SQUASH).^21^ Moderate‐to‐vigorous physical activity (hours/week) included all activities with a metabolic equivalent value ≥3 according to Ainsworth et al.^22^ Clinical data, such as disease stage (I–III), tumour location (colon: caecum to the sigmoid colon; rectum: rectosigmoid junction and rectum) and type of treatment (only surgery, surgery and chemotherapy, surgery and radiotherapy, surgery and chemotherapy and radiotherapy) were collected via the Dutch ColoRectal Audit (COLON)^23^ or the Netherlands Cancer Registry (PLCRC‐PROTECT). To assess possible confounding by other dietary factors previously associated with CRC risk or prognosis, we also calculated total intake of wholegrains, carbohydrates, dietary fibre, low‐fat dairy, high‐fat dairy and alcohol. Low‐fat dairy included low‐fat or skimmed versions of milk, yoghurt, custard and soft curd cheese. High‐fat dairy included whole‐fat versions of milk, yoghurt, custard, soft curd cheese and all other cheeses, condensed milk, ice cream, whipped cream and butter.

### 2.4 Assessment of outcome

CRC recurrences were defined as a locoregional recurrence and/or metastasis. Locoregional recurrence was defined as a CRC recurrence in the same segment as the primary tumour, in the lymph nodes of the same segment, or in the draining lymph nodes. For both cohorts, recurrence data were collected from medical charts according to standardized procedures of trained data managers of the Netherlands Comprehensive Cancer Organisation and provided via the Netherlands Cancer Registry and most recently updated in July 2022 (COLON) and July 2024 (PLCRC‐PROTECT). All‐cause mortality data were obtained from the municipal registrations, most recently updated in June 2023 (COLON) and January 2023 (PLCRC‐PROTECT).

For recurrence analyses dedicated to pre‐diagnostic intake, follow‐up time was calculated from the date of surgery, as recurrence—by definition—cannot occur before the primary tumour resection. For post‐diagnostic intake analyses, the date of filling out the post‐diagnostic questionnaires was used as the start date. Follow‐up time ended at the date of recurrence, until the date recurrence status was updated, or until the end of follow‐up (e.g., due to death, occurrence of another primary tumour with metastasis, or moving abroad), whichever came first. For analyses with all‐cause mortality as the outcome measure, follow‐up time was calculated starting from the date of surgery (for pre‐diagnostic intake) or the date of filling out the questionnaires (for post‐diagnostic intake) until the date of death, until the date the vital status was last updated, or until the end of follow‐up, whichever came first. If no surgery was performed or if the date of surgery was unavailable, the date of filling out the FFQ (*n* = 46) or the date of filling out the general questionnaire (*n* = 1) was used. If the date of filling out the post‐diagnostic FFQ was unavailable, the date of filling out the post‐diagnostic general questionnaire was used (*n* = 34).

### 2.5 Data analysis

Sex‐specific quartiles of meat intake were constructed. Population characteristics are presented as medians (interquartile range [IQR]) or numbers (percentage).

Cox proportional hazards regression analyses were used to calculate HRs and 95% CIs for the associations between pre‐ and 6‐month post‐diagnostic intake of total meat, processed meat, unprocessed red meat and unprocessed poultry and risk of recurrence and all‐cause mortality, in the total study population and in strata of sex (female/male), primary tumour location (colon/rectum) and disease stage at diagnosis (I–II/III). Log–log curves were visually inspected for non‐parallelism to check the proportionality assumption for the Cox proportional hazards model, and the assumption was met.

First, a crude model was created, adjusting for age, sex (except for analyses in strata of sex) and total energy intake. Then, potential confounders were added to the model to assess whether they changed the HR. The following covariates were considered as confounders based on literature: primary tumour location (proximal colon, distal colon, rectum; except for analyses in strata of primary tumour location), disease stage (I + II, III; except for analyses in strata of disease stage), BMI (continuous in kg/m^2^), education level (low, medium, high), smoking status (current, former, never), moderate‐to‐vigorous physical activity (continuous in hours/week) and total dietary intake of wholegrains, dietary fibre, carbohydrates, low‐fat dairy, high‐fat dairy, coffee and alcohol (all continuous in g/day). After inclusion of these confounders one by one, the HRs changed by 0–10%, with the majority of variables changing the HRs with 0–3%. Based on these results, we opted to include covariates that changed the HR by at least 7%. As a result, the fully adjusted model included age, sex, education level, disease stage (except for analyses stratified by disease stage), primary tumour location (except for analyses stratified by primary tumour location) and total daily intakes of energy, low‐fat dairy and high‐fat dairy. Analyses were not adjusted for type of treatment, as this strongly depends on stage of disease, so additionally adjusting for type of treatment would result in multicollinearity. Also, post‐diagnostic meat intake in participants with adjuvant chemotherapy (processed meat: 26 g/day, unprocessed red meat: 31 g/day, unprocessed poultry: 10 g/day) was similar to post‐diagnostic intake in the total study population (processed meat: 25 g/day, unprocessed red meat: 31 g/day, unprocessed poultry: 10 g/day), suggesting that type of treatment (here chemotherapy) did not substantially impact the exposure (meat intake).

In the post‐diagnostic analyses with mortality as the outcome, we applied stabilised inverse probability weighting to avoid survivorship bias, as participants who survived long enough to provide post‐diagnostic FFQ data may differ from participants who died before the post‐diagnostic measurement.^24^ The propensity scores were estimated based on age, sex, stage (stage I, stage II, stage III) and primary tumour location (proximal colon, distal colon, rectum). Differences with non‐weighted analyses were negligible and did not change conclusions.

Associations were visualised using restricted cubic splines with the fully adjusted model. Based on Akaike's information criterion, the model was observed to fit best with 3 knots for analyses with processed meat and unprocessed poultry, and knots were placed at the 5th, 50th, and 95th percentiles. The model was observed to fit best with 4 knots for analyses with unprocessed red meat, and knots were placed at the 5th, 35th, 65th, and 95th percentiles. Data points below the 1st and above the 99th percentiles were omitted, and graphs were manually truncated for layout purposes at 80 g/day for processed meat and unprocessed red meat, and at 40 g/day for unprocessed poultry intakes (not displaying up to 4.8% of observations in the spline plot). A meat intake of 0 g/day was set as the reference. For non‐linear associations, the lowest HR, 95% CI and accompanying level of intake were reported in the spline. For those associations that were not significantly non‐linear, we added HRs for 50 g/day increments to the spline plots in accordance with portion sizes.

Sensitivity analyses were performed mutually adjusting for other meat types. Besides, as the aetiology and prognosis may be different for early‐onset CRC,^25^ we also performed sensitivity analyses excluding participants who were younger than 50 years old at their diagnosis (*n* = 128). To assess potential reverse causality, we also excluded those with a recurrence or those who died within 6 months after surgery (*n* = 21 recurrences and *n* = 20 deaths) or within 6 months after the post‐diagnostic FFQ (*n* = 62 recurrences and *n* = 9 deaths) in sensitivity analyses.

Data analyses were performed using R Statistical Software (version 4.3.1). *p*‐values below 0.05 were considered statistically significant.

## 3 RESULTS

For pre‐diagnostic analyses with recurrence as outcome, 336 events occurred during a median follow‐up time of 5.0 (IQR: 3.1, 7.3) years. For post‐diagnostic analyses with recurrence as outcome, 275 events occurred during a median follow‐up time of 4.8 (IQR: 3.0, 7.0) years. For pre‐diagnostic analyses with all‐cause mortality as outcome, 409 events occurred during a median follow‐up time of 6.4 (IQR: 4.3, 8.4) years. For post‐diagnostic analyses with all‐cause mortality as outcome, 336 events occurred during a median follow‐up time of 6.4 (IQR: 4.1, 8.1) years.

Thirty‐eight per cent of participants were female, and participants were on average 66 years old (IQR: 60, 72) (Table 1). Participants in the upper quartiles of processed meat and unprocessed red meat intake at diagnosis had a higher BMI, were less often highly educated, were slightly more physically active, and more often received (neo‐)adjuvant treatment, compared to participants in the lower quartiles of processed meat and unprocessed red meat intake, respectively. Also, participants in the upper versus lower quartiles of processed meat and unprocessed red meat intake consumed more energy, coffee and alcohol. Additionally, participants in the upper quartile of processed meat intake consumed more wholegrains, unprocessed red meat and total saturated fatty acids, compared to participants in the lower quartiles. Participants in the upper quartile of unprocessed red meat intake were less often never smokers and consumed more processed meat and poultry, compared to participants in the lower quartile. Participants in the upper quartile of unprocessed poultry intake were younger, less often low‐educated, more often former smokers, more often had stage III disease and less often had a tumour in the proximal colon, compared to participants in the lower quartile of unprocessed poultry intake. Also, participants in the upper quartile of unprocessed poultry intake consumed more unprocessed red meat, low‐fat dairy and coffee, and consumed less high‐fat dairy.

**TABLE 1:** Baseline characteristics of the study population by sex‐specific quartiles of processed meat, unprocessed red meat and unprocessed poultry intake at diagnosis.

Characteristics	Total population (*n* = 2336)	Sex‐specific quartile of meat intake					
		Processed meat		Unprocessed red meat		Unprocessed poultry	
		Q1 (*n* = 584)	Q4 (*n* = 585)	Q1 (*n* = 584)	Q4 (*n* = 585)	Q1 (*n* = 576)	Q4 (*n* = 586)
Age, years	65.8 [60.0, 71.7]	65.9 [60.5, 71.0]	65.6 [60.0, 71.1]	65.8 [60.2, 71.7]	66.1 [60.0, 72.1]	67.4 [62.6, 73.2]	63.4 [57.5, 69.8]
Female	877 (37.5)	219 (37.5)	220 (37.6)	219 (37.5)	220 (37.6)	216 (37.5)	220 (37.5)
BMI, kg/m^2^	26.0 [23.9, 28.7]	25.2 [23.0, 27.7]	27.0 [24.4, 30.0]	25.4 [23.4, 28.1]	26.6 [24.3, 29.4]	25.9 [23.6, 28.4]	26.4 [24.1, 29.4]
Waist‐hip‐ratio	0.95 [0.90, 1.01]	0.95 [0.89, 1.00]	0.96 [0.90, 1.02]	0.95 [0.89, 1.00]	0.96 [0.91, 1.02]	0.95 [0.90, 1.00]	0.95 [0.90, 1.00]
Level of education^a^							
Low	919 (39.3)	177 (30.3)	279 (47.7)	203 (34.8)	267 (45.6)	244 (42.4)	196 (33.4)
Medium	609 (26.1)	151 (25.9)	149 (25.5)	141 (24.1)	147 (25.1)	138 (24.0)	179 (30.5)
High	800 (34.2)	254 (43.5)	154 (26.3)	238 (40.8)	169 (28.9)	190 (33.0)	208 (35.5)
Unknown	8 (0.3)	2 (0.3)	3 (0.5)	2 (0.3)	2 (0.3)	4 (0.7)	3 (0.5)
Smoking status							
Current	213 (9.1)	43 (7.4)	65 (11.1)	42 (7.2)	65 (11.1)	56 (9.7)	46 (7.8)
Former	1348 (57.7)	329 (56.3)	330 (56.4)	310 (53.1)	347 (59.3)	311 (54.0)	351 (59.9)
Never	734 (31.4)	205 (35.1)	178 (30.4)	219 (37.5)	165 (28.2)	194 (33.7)	174 (29.7)
Unknown	41 (1.8)	7 (1.2)	12 (2.1)	13 (2.2)	8 (1.4)	15 (2.6)	15 (2.6)
Moderate‐to‐vigorous physical activity, hours/week^b^	11.0 [5.3, 19.5]	10.8 [5.1, 18.5]	11.5 [5.1, 19.5]	10.6 [5.5, 18.9]	11.5 [5.1, 20.0]	11.1 [5.1, 20.0]	10.8 [5.2, 19.0]
Dietary intake							
Total energy, kcal/day	1796 [1481, 2160]	1663 [1331, 2056]	1998 [1695, 2379]	1707 [1362, 2070]	1919 [1554, 2324]	1744 [1388, 2114]	1834 [1530, 2185]
Processed meat, g/day	27 [13, 44]	5 [1, 10]	59 [50, 74]	17 [5, 35]	32 [18, 49]	22 [8, 38]	25 [12, 44]
Unprocessed red meat, g/day	34 [20, 48]	23 [8, 39]	38 [26, 53]	11 [3, 16]	61 [53, 71]	23 [6, 44]	38 [26, 56]
Unprocessed poultry, g/day	10 [4, 16]	9 [2, 18]	10 [6, 16]	5 [1, 11]	13 [8, 20]	0 [0, 3]	25 [20, 33]
Fish, g/day	11 [5, 17]	10 [4, 18]	11 [5, 17]	11 [4, 18]	9 [4, 15]	9 [2, 15]	11 [5, 17]
Low‐fat dairy, g/day^c^	161 [69, 279]	161 [60, 279]	171 [68, 290]	161 [62, 279]	157 [63, 279]	150 [54, 276]	183 [75, 286]
High‐fat dairy, g/day^d^	73 [39, 131]	74 [37, 128]	78 [43, 130]	71 [38, 128]	73 [39, 132]	82 [44, 148]	66 [36, 123]
Dietary fibre, g/day	20 [16, 24]	19 [15, 24]	21 [17, 25]	20 [16, 25]	20 [16, 24]	19 [15, 24]	20 [16, 24]
Wholegrains, g/day	108 [73, 149]	98 [65, 144]	124 [87, 162]	105 [69, 154]	106 [72, 141]	104 [69, 143]	108 [73, 151]
Total protein, g/day	69 [58, 83]	63 [50, 75]	78 [67, 93]	61 [51, 75]	77 [65, 92]	63 [52, 76]	74 [63, 88]
Animal protein, g/day	41 [33, 50]	35 [26, 43]	49 [40, 59]	33 [26, 42]	49 [42, 59]	35 [27, 44]	46 [39, 56]
Total carbohydrates, g/day	192 [156, 233]	182 [145, 224]	204 [169, 249]	188 [151, 226]	196 [160, 241]	191 [150, 228]	188 [156, 229]
Coffee, g/day	348 [232, 580]	348 [232, 522]	464 [241, 580]	348 [232, 522]	464 [232, 580]	348 [232, 537]	464 [232, 580]
Alcohol, g/day	8 [1, 20]	5 [0, 19]	9 [1, 21]	5 [0, 18]	10 [1, 24]	6 [0, 19]	8 [1, 21]
Saturated fat, g/day	25 [19, 32]	22 [16, 29]	29 [23, 35]	23 [17, 30]	26 [20, 34]	24 [18, 31]	25 [19, 32]
Heme iron, mg/day	0.8 [0.6, 1.1]	0.5 [0.3, 0.7]	1.1 [0.9, 1.3]	0.4 [0.3, 0.6]	1.1 [1.0, 1.4]	0.6 [0.3, 0.9]	0.9 [0.7, 1.2]
Clinical characteristics							
Location of the tumour^e^							
Colon	1565 (67.0)	383 (65.6)	386 (66.0)	405 (69.3)	402 (69.2)	390 (67.7)	390 (66.6)
Rectum	769 (32.9)	201 (34.4)	199 (34.0)	179 (30.7)	182 (31.1)	186 (32.3)	196 (33.4)
Unknown	2 (0.1)	0 (0.0)	0 (0.0)	0 (0.0)	1 (0.2)	0 (0.0)	0 (0.0)
Disease stage							
I	661 (28.3)	158 (27.1)	160 (27.4)	170 (29.1)	142 (24.3)	174 (30.2)	164 (28.0)
II	645 (27.6)	160 (27.4)	157 (26.8)	162 (27.7)	171 (29.2)	165 (28.6)	156 (26.6)
III	1030 (44.1)	266 (45.5)	268 (45.8)	252 (43.2)	272 (46.5)	237 (41.1)	266 (45.4)
Type of treatment^f^							
No surgery or unknown	39 (1.7)	9 (1.5)	8 (1.4)	12 (2.1)	8 (1.4)	12 (2.1)	11 (1.9)
Surgery only	1299 (55.6)	334 (57.2)	302 (51.6)	339 (58.0)	313 (53.5)	342 (59.4)	325 (55.5)
Surgery + chemotherapy	557 (23.8)	131 (22.4)	146 (25.0)	124 (21.2)	155 (26.5)	120 (20.8)	141 (24.1)
Surgery + radiotherapy	230 (9.8)	53 (9.1)	67 (11.5)	57 (9.8)	58 (9.9)	52 (9.0)	64 (10.9)
Surgery + chemotherapy + radiotherapy	211 (9.0)	57 (9.8)	62 (10.6)	52 (8.9)	51 (8.7)	50 (8.7)	45 (7.7)

*Note*: Values are presented as median [IQR] or number (percentage).

Abbreviation: IQR, interquartile range.

^a^ Low education was defined as primary school and lower general secondary education; medium as lower vocational training and higher general secondary education; high as higher vocational training and university.

^b^ Moderate‐to‐vigorous physical activity included all activities with a metabolic equivalent value ≥3.^22^ Data were missing for 97 participants.

^c^ Low‐fat dairy included low‐fat or skimmed versions of milk, yogurt, custard and soft curd cheese.

^d^ High‐fat dairy included whole‐fat versions of milk, yogurt, custard, soft curd cheese and all other cheeses, condensed milk, ice cream, whipped cream and butter.

^e^ Proximal colon includes the caecum, appendix, ascending colon, hepatic flexure and transverse colon. Distal colon includes the splenic flexure, descending colon and sigmoid colon. Rectum includes the rectosigmoid junction and rectum.

^f^ Treatment includes neoadjuvant and adjuvant treatment.

Median pre‐diagnostic intakes of processed meat, unprocessed red meat and unprocessed poultry were 27 (IQR: 13, 44) g/day, 34 (IQR: 20, 48) g/day and 10 (IQR: 4, 16) g/day, respectively. Median post‐diagnostic intakes of processed meat, unprocessed red meat, and unprocessed poultry were 25 (IQR: 12, 41) g/day, 31 (IQR: 18, 44) g/day and 10 (IQR: 4, 17) g/day, respectively. The median change between pre‐ and post‐diagnostic intakes was −1 (IQR: −12, 9) g/day, −2 (IQR: −14, 9) g/day, and 0 (IQR: −5, 5) g/day, respectively, for those with dietary data available at both timepoints (*n* = 2002).

**FIGURE 2:**
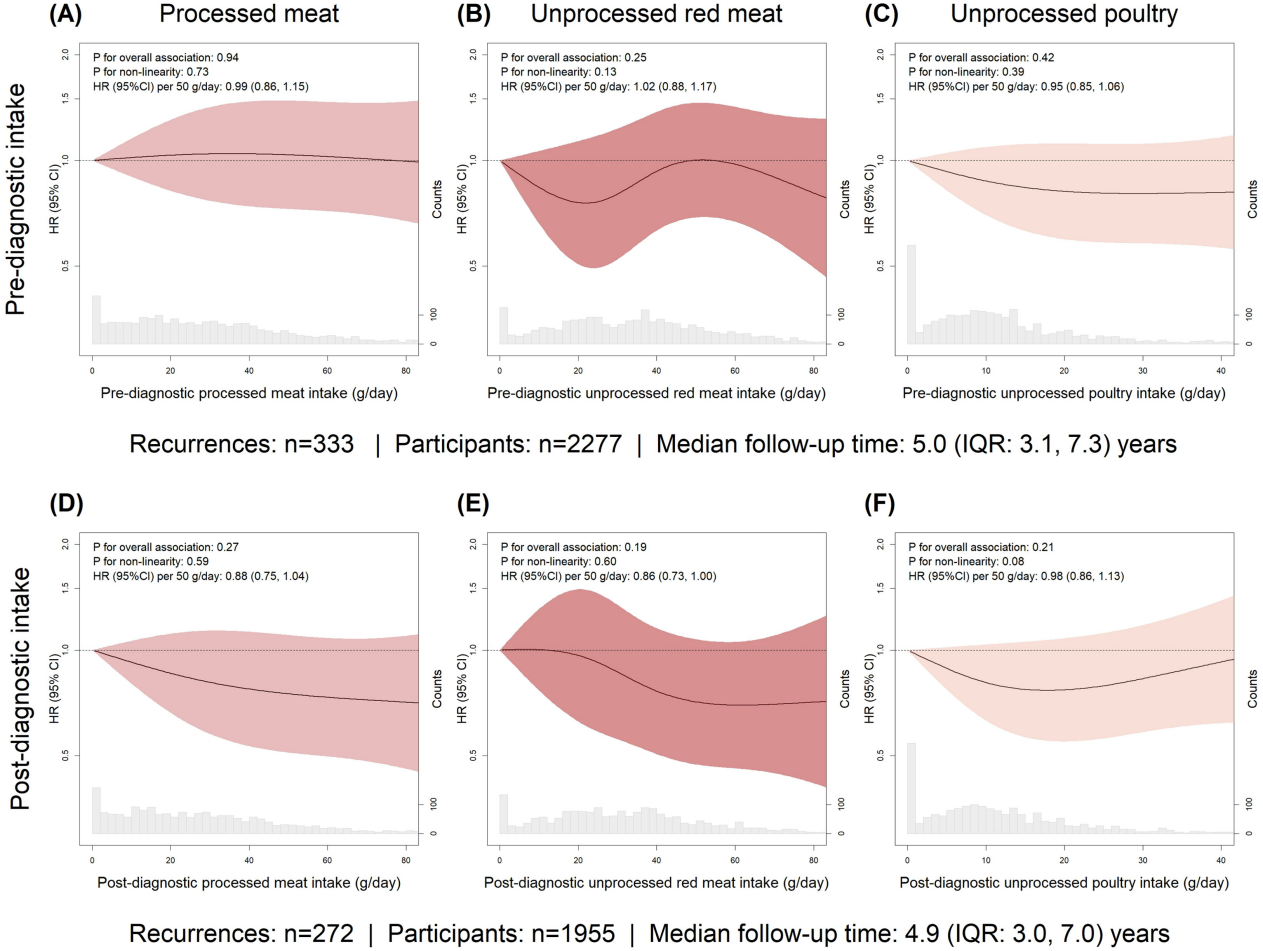
Pre‐ and post‐diagnostic intake of processed meat (A, D), unprocessed red meat (B, E) and unprocessed poultry (C, F) in relation to risk of recurrence. The fully adjusted model included age, sex, education level, disease stage, primary tumour location and total daily intakes of energy, low‐fat dairy, and high‐fat dairy.

**FIGURE 3:**
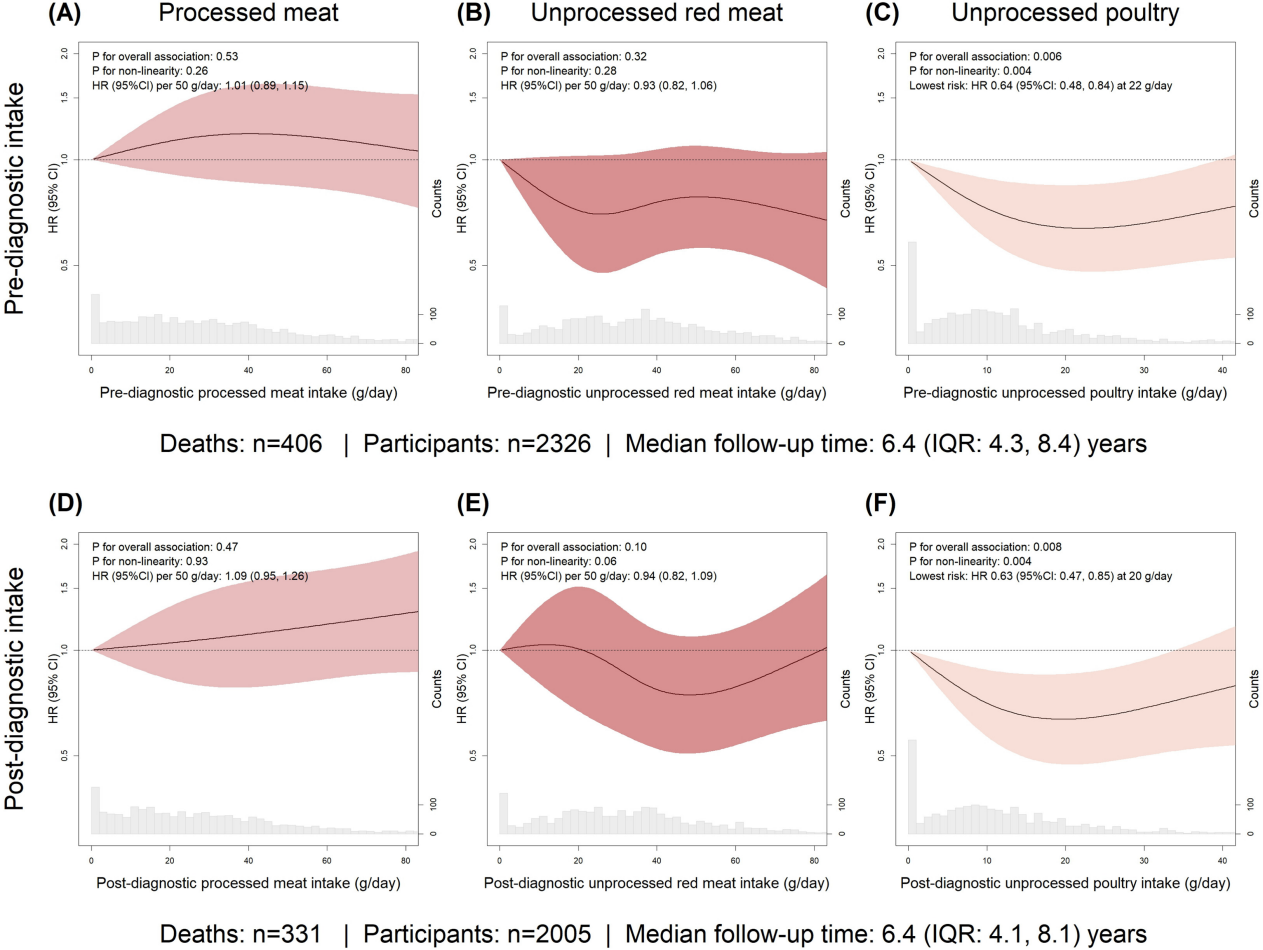
Pre‐ and post‐diagnostic intake of processed meat (A, D), unprocessed red meat (B, E) and unprocessed poultry (C, F) in relation to risk of all‐cause mortality. The fully adjusted model included age, sex, education level, disease stage, primary tumour location and total daily intakes of energy, low‐fat dairy and high‐fat dairy.

In the total study population, pre‐ and post‐diagnostic intakes of processed meat and unprocessed red meat were not associated with risk of recurrence or all‐cause mortality (Figures 2 and 3). Unprocessed poultry intake was not associated with risk of recurrence (Figure 2). A higher intake of unprocessed poultry, both before and after diagnosis, was associated with a reduced risk of all‐cause mortality (before diagnosis: P for overall association: 0.006, P for non‐linearity: 0.004; after diagnosis: P for overall association: 0.008, P for non‐linearity: 0.004; Figure 3). These associations were not affected by mutual adjustment of processed meat and unprocessed red meat intakes in sensitivity analyses (Figure S8). Compared to 0 g/day of post‐diagnostic unprocessed poultry intake, the lowest risk of all‐cause mortality was observed at 20 g/day with an HR of 0.63 (95% CI: 0.47, 0.85; Figure 3).

**FIGURE 4:**
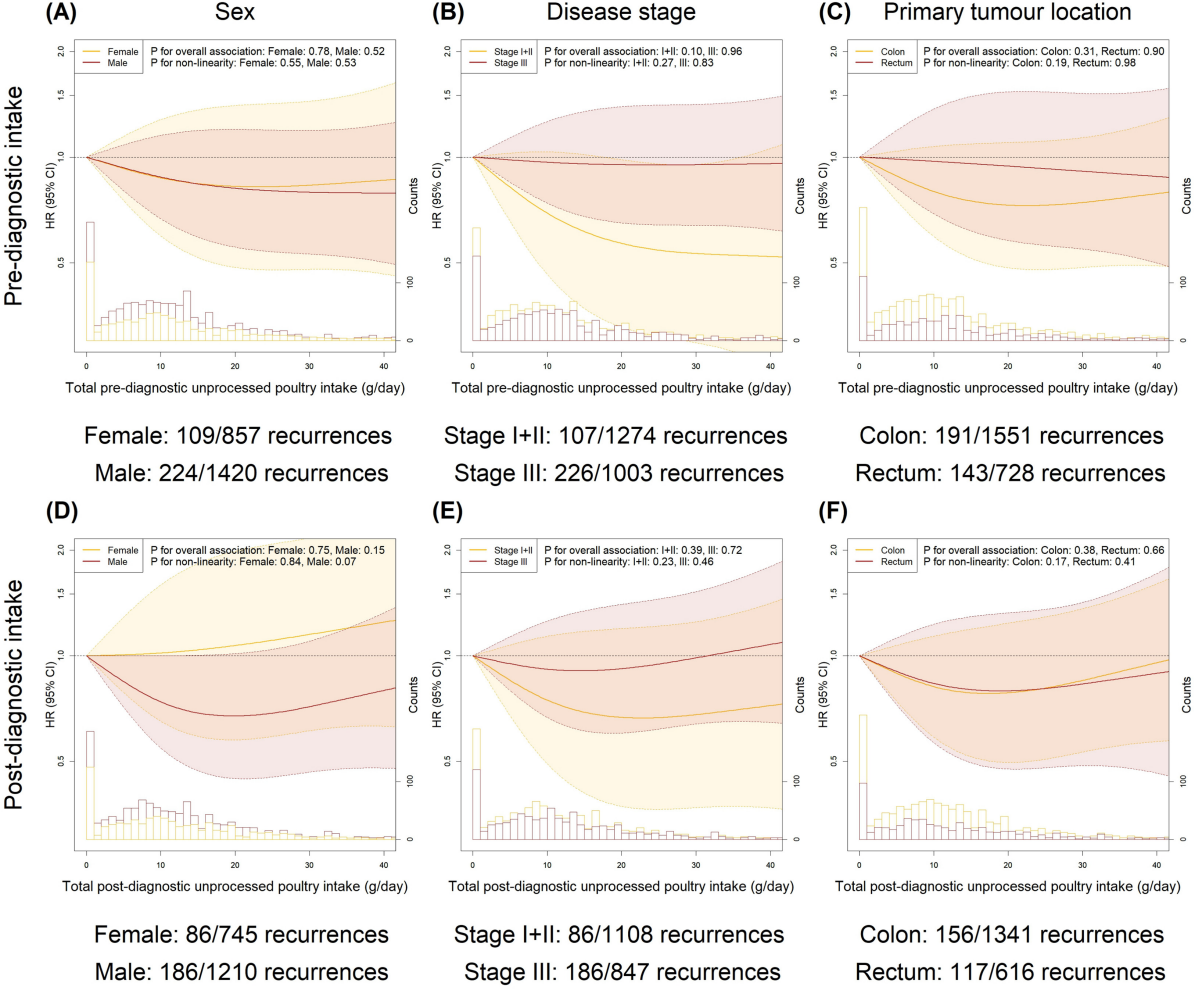
Pre‐ and post‐diagnostic intakes of unprocessed poultry in relation to risk of recurrence stratified by sex (A, D), disease stage (B, E) and primary tumour location (C, F). The fully adjusted model included age, sex (except for analyses stratified by sex), education level, disease stage (except for analyses stratified by disease stage), primary tumour location (except for analyses stratified by primary tumour location) and total daily intakes of energy, low‐fat dairy and high‐fat dairy. The numbers presented under each figure represent the number of recurrences in this subgroup and the total number of participants in this subgroup. The median follow‐up times (interquartile range [IQR]) were as follows for analyses concerning pre‐diagnostic intake: female: 5.0 [3.2, 7.5] years; male: 5.0 [2.9, 7.0] years; stage I + II: 5.4 [3.5, 7.4] years; stage III: 4.8 [2.5, 6.9] years; colon: 5.1 [3.2, 7.4] years; rectum: 4.8 [2.6, 7.2] years. The median follow‐up times [IQR] were as follows for analyses concerning post‐diagnostic intake: Female: 4.8 [3.1, 7.1] years; male: 4.8 [2.8, 7.0] years; stage I + II: 5.1 [3.2, 7.1] years; stage III: 4.5 [2.1, 7.0] years; colon: 4.9 [3.0, 7.0] years; rectum: 4.7 [2.9, 7.0] years.

**FIGURE 5:**
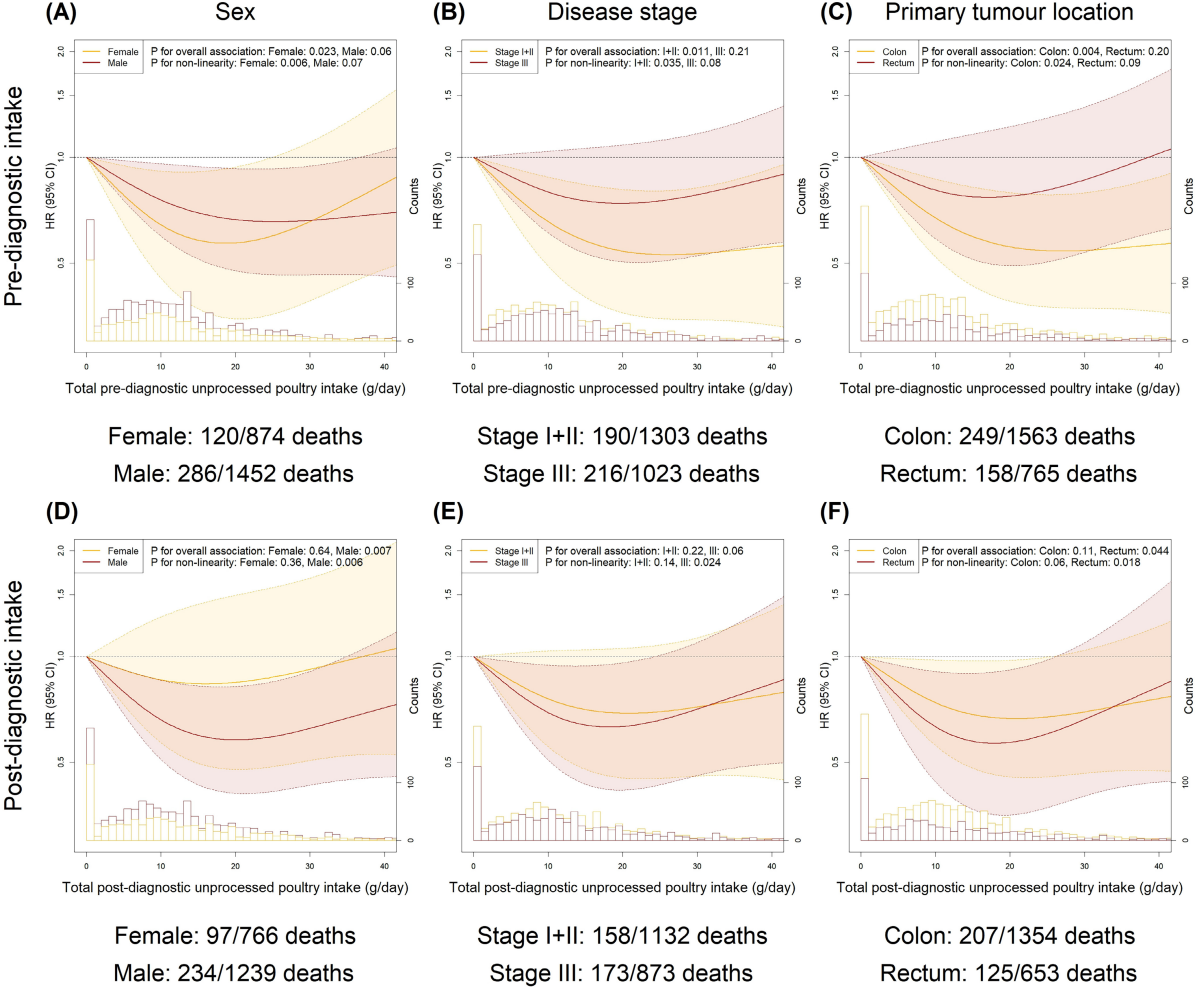
Pre‐ and post‐diagnostic intakes of unprocessed poultry in relation to the risk of all‐cause mortality stratified by sex (A, D), disease stage (B, E) and primary tumour location (C, F). The fully adjusted model included age, sex (except for analyses stratified by sex), education level, disease stage (except for analyses stratified by disease stage), primary tumour location (except for analyses stratified by primary tumour location) and total daily intakes of energy, low‐fat dairy and high‐fat dairy. The numbers presented under each figure represent the number of deaths in this subgroup and the total number of participants in this subgroup. The median follow‐up times [IQR] were as follows for analyses concerning pre‐diagnostic intake: female: 6.4 [4.4, 8.7] years; male: 6.4 [4.2, 8.3] years; stage I + II: 6.5 [4.5, 8.5] years; stage III: 6.2 [4.0, 8.3] years; colon: 6.4 [4.3, 8.4] years; rectum: 6.3 [4.2, 8.4] years. The median follow‐up times [IQR] were as follows for analyses concerning post‐diagnostic intake: Female: 6.4 [4.1, 8.4] years; male: 6.3 [4.0, 8.0] years; stages I + II: 6.5 [4.2, 8.2] years; stage III: 6.2 [3.8, 8.1] years; colon: 6.4 [4.1, 8.1] years; rectum: 6.4 [4.1, 8.1] years.

In subgroup analyses, we observed no substantially different associations between meat intakes and risk of recurrence and all‐cause mortality for subgroups of sex, disease stage, or primary tumour location given overlaps in the corresponding 95% CIs (Figures 4 and 5 for unprocessed poultry; Figures S1–S4 for processed meat and unprocessed red meat).

None of the sensitivity analyses markedly affected the main results in the overall study population (Figures S5–S10). Neither did mutual adjustment for other meat types affect results in subgroup analyses (data not shown). Excluding participants who were younger than 50 years old at their diagnosis (*n* = 128) or excluding those with an event within 6 months after surgery (*n* = 21 recurrences and *n* = 20 deaths) or within 6 months after the post‐diagnostic FFQ (*n* = 62 recurrences and *n* = 9 deaths) did not affect results in subgroup analyses (data not shown).

## 4 DISCUSSION

In the current study, higher intakes of both pre‐ and post‐diagnostic unprocessed poultry were associated with a decreased risk of all‐cause mortality, but not with CRC recurrence. Processed meat and unprocessed red meat intakes were not associated with the risk of recurrence or all‐cause mortality. In general, subgroup analyses revealed no consistent differences based on sex, disease stage and primary tumour location.

As far as we know, only one other study assessed the relation between poultry intake and risk of all‐cause mortality in people with CRC,^26^ and none have studied unprocessed poultry or CRC recurrence as outcome measure. The EPIC cohort included 1603 males and 2186 females with CRC from 10 European countries and demonstrated a borderline statistically significant decreased risk of all‐cause mortality with higher pre‐diagnostic poultry intakes in females (HR_per 20 g/day_: 0.92, 95% CI: 0.84, 1.00), but not in males (HR_per20g/day_: 1.00, 95% CI: 0.91, 1.09).^26^ In our subgroup analyses, associations between pre‐diagnostic unprocessed poultry intake and all‐cause mortality were similar for females and males, although risk estimates were of smaller size and did not reach statistical significance in males. In both the current study and the EPIC cohort, a significantly decreased risk of all‐cause mortality has been observed with ~ 20 g/day of poultry intake, indicating the required portion size for potential health benefit is reasonable. Future studies in individuals with CRC should focus on unprocessed poultry intake and confirm that potential health benefits can be achieved by consuming ~ 20 g/day of unprocessed poultry.

In line with findings in the CRC population, a recent meta‐analysis including 24 cohort studies in the general population demonstrated that a higher poultry intake was associated with a small but statistically significantly reduced risk of all‐cause mortality (highest versus lowest intake category: risk ratio: 0.96; 95% CI: 0.93, 0.98).^27^ In the same meta‐analysis, substitution analyses showed a significantly decreased risk of cardiovascular disease when substituting processed meat with poultry (RR_per 100 g/day substitution_: 0.71; 95% CI: 0.56, 0.90). Similarly, replacing total red meat with poultry was associated with a reduced risk of coronary heart disease (RR_per 100 g/day substitution_: 0.83; 95% CI: 0.72, 0.95).^27^ Substituting total red meat (RR_per 100 g/day substitution_: 0.88; 95% CI: 0.78, 0.99), unprocessed red meat (RR_per 100 g/day substitution_: 0.87; 95% CI: 0.82, 0.92), and processed meat (RR_per 100 g/day substitution_: 0.84; 95% CI: 0.78, 0.92) with poultry was associated with a reduced risk of all‐cause mortality.^27^ It could be hypothesised that substitution of processed meat and unprocessed red meat with poultry leads to a lower heme iron intake, which could lead to a reduced stimulation of the endogenous production of carcinogenic *N*‐nitroso compounds,^1^, ^9^ and reduced damage by heme to colonic epithelial cells.^10^ In our study, participants in the upper quartile of poultry intake also had a higher intake of processed meat, unprocessed red meat and total heme iron, compared to those in the lowest quartile (Table 1). Therefore, in our study, poultry does not necessarily seem to be consumed as a substitute for processed meat and unprocessed red meat. Also, even though poultry contains a lower amount of heme iron than red meat, in the current study, total heme iron intake in the highest quartile of poultry intake was comparable to that of the highest quartile of unprocessed red meat intake. Therefore, in the current study, the reduced risk of all‐cause mortality in those individuals with high poultry intakes, compared to individuals with a low poultry intake, does not seem to be explained by a reduced heme iron intake.

As we observed a higher poultry intake to be associated with a lower risk of all‐cause mortality, but not CRC recurrence specifically, incidence or progression of other chronic diseases such as cardiovascular disease may play an important role in the observed association. Moreover, people who regularly consume poultry may also behave differently in other lifestyle domains, compared to those who do not regularly consume poultry (Table 1). We assessed the impact of including BMI, education level, smoking status and moderate‐to‐vigorous physical activity in the models. Only education level substantially impacted associations and was therefore kept in the model. Other or related factors, such as health consciousness and socioeconomic status, could still partially explain the observed association between poultry consumption and all‐cause mortality risk. It could be that other factors associated with poultry intake, such as health consciousness and a generally healthier lifestyle, partially explain the observed association between poultry intake and all‐cause mortality risk.

In contrast to consistent associations with CRC risk,^1^ we observed no associations between pre‐ and post‐diagnostic processed meat and unprocessed red meat intakes in relation to the CRC recurrence or all‐cause mortality risk, in the total study population nor in subgroup analyses. Our findings are mostly in line with those of a recent pooled analysis of 10 studies, which included 7627 participants with stage I–III CRC. In this pooled analysis, pre‐diagnostic red and processed meat intakes were also not associated with the risk of all‐cause mortality,^2^ except for when comparing participants with a higher versus lower processed meat intake than the median intake (HR: 1.12, 95% CI: 1.01, 1.25). In the current study, participants with a processed meat intake higher than the median intake had no significantly higher risk of all‐cause mortality than those with a processed meat intake below the median intake (HR: 1.08, 95%CI: 0.88, 1.32).

We observed no consistent differences in the associations for subgroups based on sex, stage and tumour location. Meat intake may, however, be differently associated with the risk of CRC recurrence or mortality by tumour mutation status.^28^ In a previous prospective cohort study in 1,245 participants with stage I–III CRC, a higher red and processed meat intake was associated with an increased risk of all‐cause mortality in participants with *KRAS*‐mutated CRC, but not in those with *KRAS*‐wild‐type CRC.^28^ Therefore, future studies might consider stratifying by the mutation status of different common tumour mutations in CRC, such as *KRAS*.

A strength of the current study is the large sample size of participants with CRC with both pre‐ and post‐diagnostic dietary intake data available. Also, the availability of recurrence data, which was retrieved in a standardised manner by specialised data managers from the national cancer registry, is a strength. A limitation of the current study is that we had no data available on cause of death because of updated privacy regulations in the Netherlands. Therefore, we cannot confirm our hypothesis that the incidence and mortality of other chronic diseases, such as cardiovascular disease, may play an important role in the association between poultry intake and all‐cause mortality risk. Future studies should include cause‐specific death data if available. Furthermore, although the use of FFQs is common in observational studies, it comes with the risk of measurement error. As the current study included participants upon diagnosis, pre‐diagnostic dietary data were assessed shortly after diagnosis, which may have induced recall error. If present, we would expect these to be non‐differential and attenuate the observed effect estimates. Also, inherent to the observational design of the study, we cannot prove causality, and there may be unmeasured confounding. Lastly, as the study populations of both cohorts are Dutch, the external validity of our findings may be limited.

To conclude, our findings suggest that a higher intake of unprocessed poultry intake is associated with a decreased risk of all‐cause mortality, while processed meat and unprocessed red meat were not associated with the risk of CRC recurrence or all‐cause mortality. Future studies in independent study populations are needed to confirm these results before they can contribute to dietary guidelines for CRC survivors.

## AUTHOR CONTRIBUTIONS

**Anne‐Sophie van Lanen:** Writing – original draft; writing – review and editing; visualization; conceptualization; investigation; formal analysis. **Dieuwertje E. Kok:** Conceptualization; writing – review and editing; funding acquisition; supervision. **Evertine Wesselink:** Conceptualization; writing – review and editing; investigation. **Jeroen W. G. Derksen:** Conceptualization; investigation; writing – review and editing. **Anne M. May:** Funding acquisition; writing – review and editing; conceptualization. **Karel C. Smit:** Conceptualization; investigation; writing – review and editing. **Miriam Koopman:** Writing – review and editing; funding acquisition; resources. **Johannes H. W. de Wilt:** Writing – review and editing; resources. **Ellen Kampman:** Writing – review and editing; conceptualization; funding acquisition; supervision. **Fränzel J. B. van Duijnhoven:** Writing – review and editing; conceptualization; funding acquisition; supervision.

## FUNDING INFORMATION

This work was financially supported by the Regio Deal Foodvalley (162135). The COLON study was financially supported by Wereld Kanker Onderzoek Fonds (WKOF) and World Cancer Research Fund International (WCRF International) as well as by funding (2014/1179, IIG_FULL_2021_022, IIG_FULL_2021_023 and IIG_FULL_2023_017) obtained from WKOF as part of the World Cancer Research Fund International grant programme; Alpe d'Huzes/Dutch Cancer Society (UM 2012‐5653, UW 2013‐5927, UW 2015‐7946); ERA‐NET on Translational Cancer Research (TRANSCAN via the Dutch Cancer Society) (UW2013‐6397, UW2014‐6877); the Netherlands Organization for Health Research and Development (ZonMw), the Netherlands); and the Regio Deal Foodvalley (162135). The Prospective Dutch Colorectal Cancer (PLCRC) cohort is an initiative of the Dutch Colorectal Cancer Group (DCCG) and is supported by the Dutch Cancer Society; Stand Up to Cancer; ZonMw; Health Holland; Maag Lever Darm Stichting; Lilly (unrestricted grant); Merck (unrestricted grant); Bristol‐Myers Squibb (unrestricted grant); Bayer (unrestricted grant); Servier (unrestricted grant); Pierre Fabre (unrestricted grant); Province of Utrecht, the Netherlands; and the Regio Deal Foodvalley (162135). The funders had no role in the design of the study; the collection, analysis, and interpretation of the data; the writing of the manuscript; and the decision to submit the manuscript for publication.

## CONFLICT OF INTEREST STATEMENT

JWGD was employed at the University Medical Center Utrecht (The Netherlands) at the time of the conduction of the study and writing of the paper, but is currently also employed at Danone Global Research and Innovation. MK reports to have institutional financial interests with Amgen, Bayer, Bristol Myers Squibb, GSK, Merck‐Serono, Nordic Farma, Personal Genome Diagnostics (PGDx), Pierre Fabre, Roche, Sirtex, Servier and Sanofi‐Aventis. MK is PI of PLCRC (national observational cohort study) and of the international cohort study PROMETCO with Servier as sponsor. MK is chair of the ESMO RWD and digital health working group and is involved in several clinical trials as PI or co‐investigator in CRC. The other co‐authors declare no conflicts of interest.

## ETHICS STATEMENT

This study was performed in line with the principles of the Declaration of Helsinki. Both the COLON study and PLCRC‐PROTECT study were approved by a medical ethics committee (COLON: region Arnhem‐Nijmegen, 2009‐349, ClinicalTrials.gov Identifier NCT03191110; PLCRC‐PROTECT: University Medical Center Utrecht, 15‐770/C, ClinicalTrials.gov Identifier NCT02070146). All study participants provided written informed consent.

## Supporting information


**Data S1.** Supporting Information.

## Data Availability

For the COLON study, requests for data can be sent to Dr. Fränzel J. B. van Duijnhoven, Division of Human Nutrition and Health, Wageningen University & Research, the Netherlands (e‐mail: franzel.vanduijnhoven@wur.nl). For the PLCRC‐PROTECT study, access to cohort resources for future collaborative research projects may be requested through the Scientific Committee of PLCRC (email: info@plcrc.nl) that reviews all research projects for approval. Further information is available from the corresponding author upon request.

## MEMBERS OF COLON AND PLCRC STUDIES

Hester van Cruijsen (Department of Medical Oncology, Antonius Hospital, Sneek, The Netherlands), Jan Willem T. Dekker (Department of Surgery, Reinier de Graaf Hospital, Delft, The Netherlands), Henk K. van Halteren (Department of Internal Medicine, Admiraal de Ruyter Hospital, Goes, The Netherlands), Johan J.B. Janssen (Department of Medical Oncology, Canisius Wilhelmina Hospital, Nijmegen, The Netherlands), Maartje Los (Department of Medical Oncology, St. Antonius Hospital, Nieuwegein, The Netherlands), Anandi H. W. Schiphorst (Department of Surgery, Diakonessenhuis Hospital, Utrecht, The Netherlands), Dirkje W. Sommeijer (Department of Internal Medicine, Flevo Hospital, Almere, The Netherlands), Dirk J. A. Sonneveld (Department of Surgery, Dijklander Hospital, Hoorn, The Netherlands), Mark P. S. Sie (Department of Medical Oncology, ZorgSaam Hospital, Terneuzen, The Netherlands), Maarten Vermaas (Department of Surgery, IJsselland Hospital, Capelle aan den IJssel, The Netherlands).
