# Supplementary material for: Pre‐ and post‐diagnostic meat intake in relation to risk of recurrence and mortality among individuals with stage I–III colorectal cancer
Source: Int J Cancer. 2025 Sep 18;158(4):895–908. doi: 10.1002/ijc.70113 (PMC12712359; doi:10.1002/ijc.70113)
Supplement: Supplementary file 1 — Data S1. Supporting Information. [file IJC-158-895-s001.pdf]

Supplementary Figures

Pre- and post-diagnostic meat intake in relation to risk of recurrence and mortality among individuals with stage I-III colorectal cancer

Anne-Sophie van Lanen, Dieuwertje E. Kok, Evertine Wesselink, Jeroen W.G. Derksen, Anne M. May, Karel C. Smit, Miriam Koopman, Johannes H. W. de Wilt, Ellen Kampman, Fränzel J.B. van Duijnhoven, on behalf of the COLON and PLCRC studies

Members of COLON and PLCRC studies: Hester van Cruijsen (Department of Medical Oncology, Antonius Hospital, Sneek, The Netherlands), Jan Willem T. Dekker (Department of Surgery, Reinier de Graaf Hospital, Delft, The Netherlands), Henk K. van Halteren (Department of Internal Medicine, Admiraal de Ruyter Hospital, Goes, The Netherlands), Johan J.B. Janssen (Department of Medical Oncology, Canisius Wilhelmina Hospital, Nijmegen, The Netherlands), Maartje Los (Department of Medical Oncology, St. Antonius Hospital, Nieuwegein, The Netherlands), Anandi H.W. Schiphorst (Department of Surgery, Diakonessenhuis Hospital, Utrecht, The Netherlands), Dirkje W. Sommeijer (Department of Internal Medicine, Flevo Hospital, Almere, The Netherlands), Dirk J.A. Sonneveld (Department of Surgery, Dijklander Hospital, Hoorn, The Netherlands), Mark P.S. Sie (Department of Medical Oncology, ZorgSaam Hospital, Terneuzen, The Netherlands), Maarten Vermaas (Department of Surgery, IJsselland Hospital, Capelle aan den IJssel, The Netherlands)

Table of contents

Part 1: Subgroup analyses for processed meat and unprocessed red meat

Fig S1 Pre- and post-diagnostic intakes of **processed meat** in relation to risk of **recurrence** stratified by sex (A, D), disease stage (B, E), and primary tumour location (C, F) 2

Fig S2 Pre- and post-diagnostic intakes of **processed meat** in relation to risk of **all-cause mortality** stratified by sex (A, D), disease stage (B, E), and primary tumour location (C, F) 3

Fig S3 Pre- and post-diagnostic intakes of **unprocessed red meat** in relation to risk of **recurrence** stratified by sex (A, D), disease stage (B, E), and primary tumour location (C, F) 4

Fig S4 Pre- and post-diagnostic intakes of **unprocessed red meat** in relation to risk of **all-cause mortality** stratified by sex (A, D), disease stage (B, E), and primary tumour location (C, F) 5

Part 2: Sensitivity analyses

Fig S5 Pre- and post-diagnostic intakes of processed meat, unprocessed red meat, and unprocessed poultry in relation to risk of **recurrence**, **when mutually adjusted for other meat types** 6

Fig S6 Pre- and post-diagnostic intakes of processed meat, unprocessed red meat, and unprocessed poultry in relation to risk of **recurrence**, **when excluding participants who were younger than 50 years old at their diagnosis (n=128)** 7

Fig S7 Pre- and post-diagnostic intakes of processed meat, unprocessed red meat, and unprocessed poultry in relation to risk of **recurrence**, **when excluding those with a recurrence within 6 months after surgery (n=21) or within 6 months after the post-diagnostic FFQ (n=62)** 8

Fig S8 Pre- and post-diagnostic intakes of processed meat, unprocessed red meat, and unprocessed poultry in relation to risk of **all-cause mortality**, **when mutually adjusted for other meat types** 9

Fig S9 Pre- and post-diagnostic intakes of processed meat, unprocessed red meat, and unprocessed poultry in relation to risk of **all-cause mortality**, **when excluding participants who were younger than 50 years old at their diagnosis (n=128)** 10

Fig S10 Pre- and post-diagnostic intakes of processed meat, unprocessed red meat, and unprocessed poultry in relation to risk of **all-cause mortality, when excluding those who died within 6 months after surgery (n=20) or within 6 months after the post-diagnostic FFQ (n=9)** 11

Part 1: Subgroup analyses for processed meat and unprocessed red meat

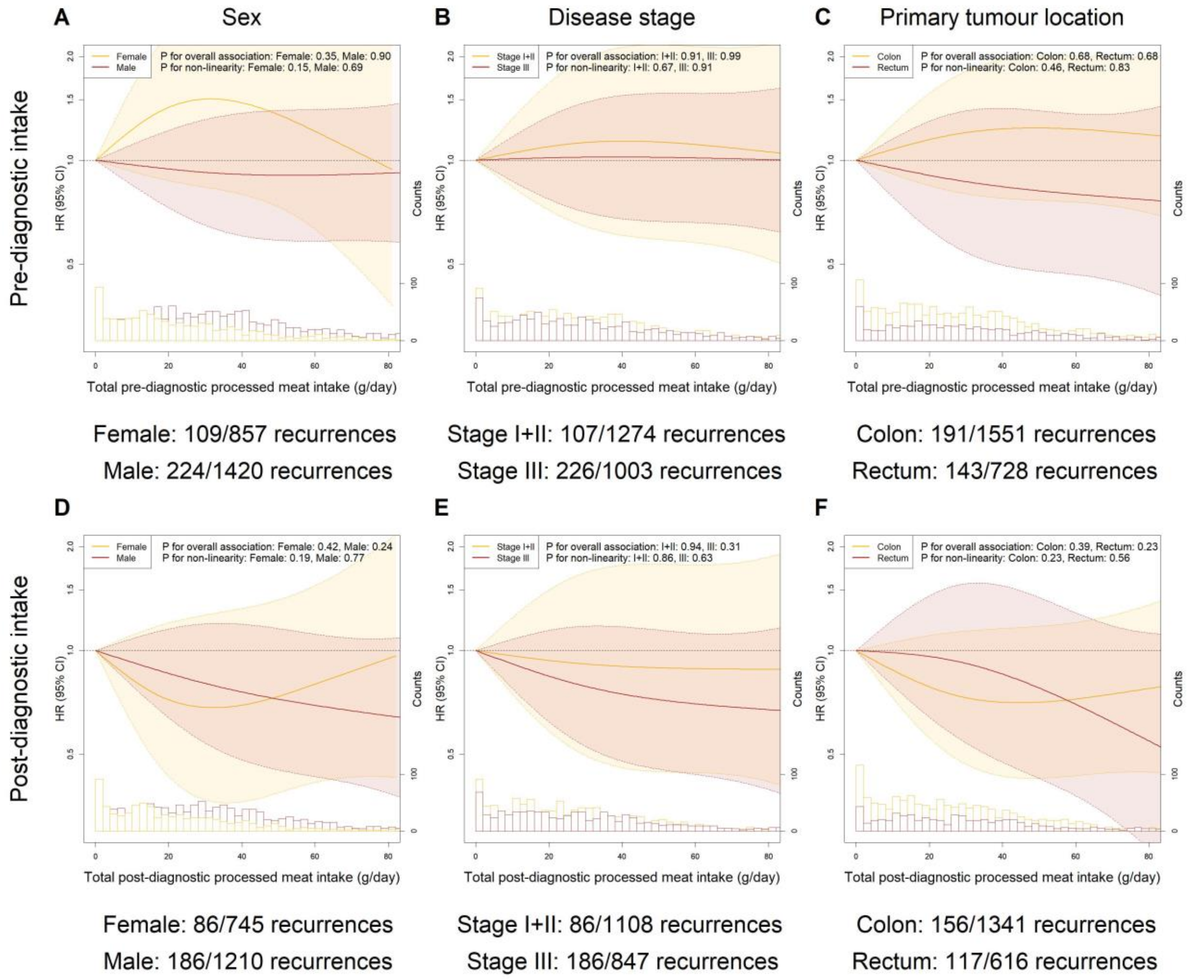

Fig S1 Pre- and post-diagnostic intakes of **processed meat** in relation to risk of **recurrence** stratified by sex (A, D), disease stage (B, E), and primary tumour location (C, F). The fully adjusted model included age, sex (except for analyses stratified by sex), education level, disease stage (except for analyses stratified by disease stage), primary tumour location (except for analyses stratified by primary tumour location), and total daily intakes of energy, low-fat dairy, and high-fat dairy. The numbers presented under each figure represent the number of recurrences in this subgroup and the total number of participants in this subgroup.

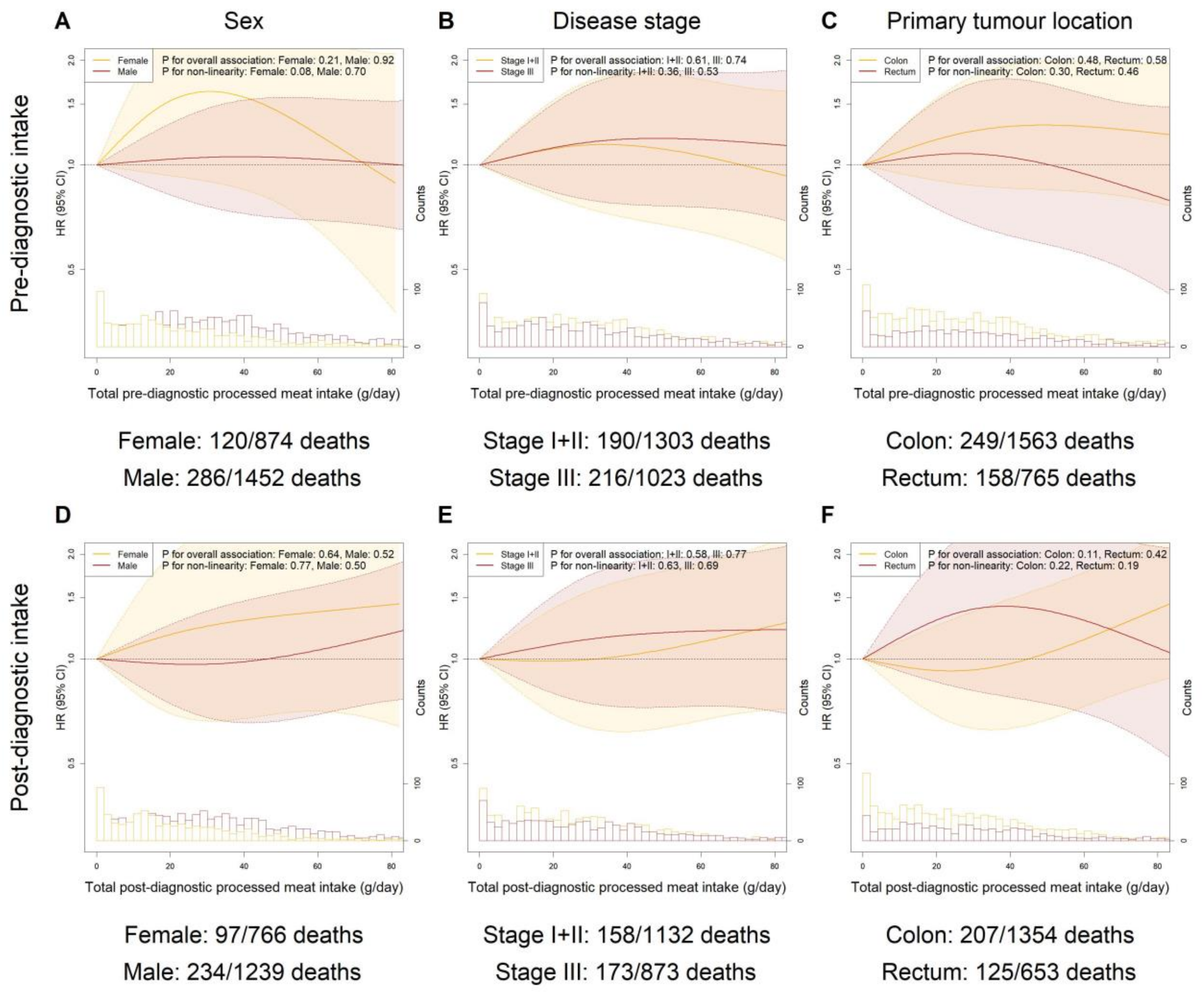

Fig S2 Pre- and post-diagnostic intakes of **processed meat** in relation to risk of **all-cause mortality** stratified by sex (A, D), disease stage (B, E), and primary tumour location (C, F). The fully adjusted model included age, sex (except for analyses stratified by sex), education level, disease stage (except for analyses stratified by disease stage), primary tumour location (except for analyses stratified by primary tumour location), and total daily intakes of energy, low-fat dairy, and high-fat dairy. The numbers presented under each figure represent the number of recurrences in this subgroup and the total number of participants in this subgroup.

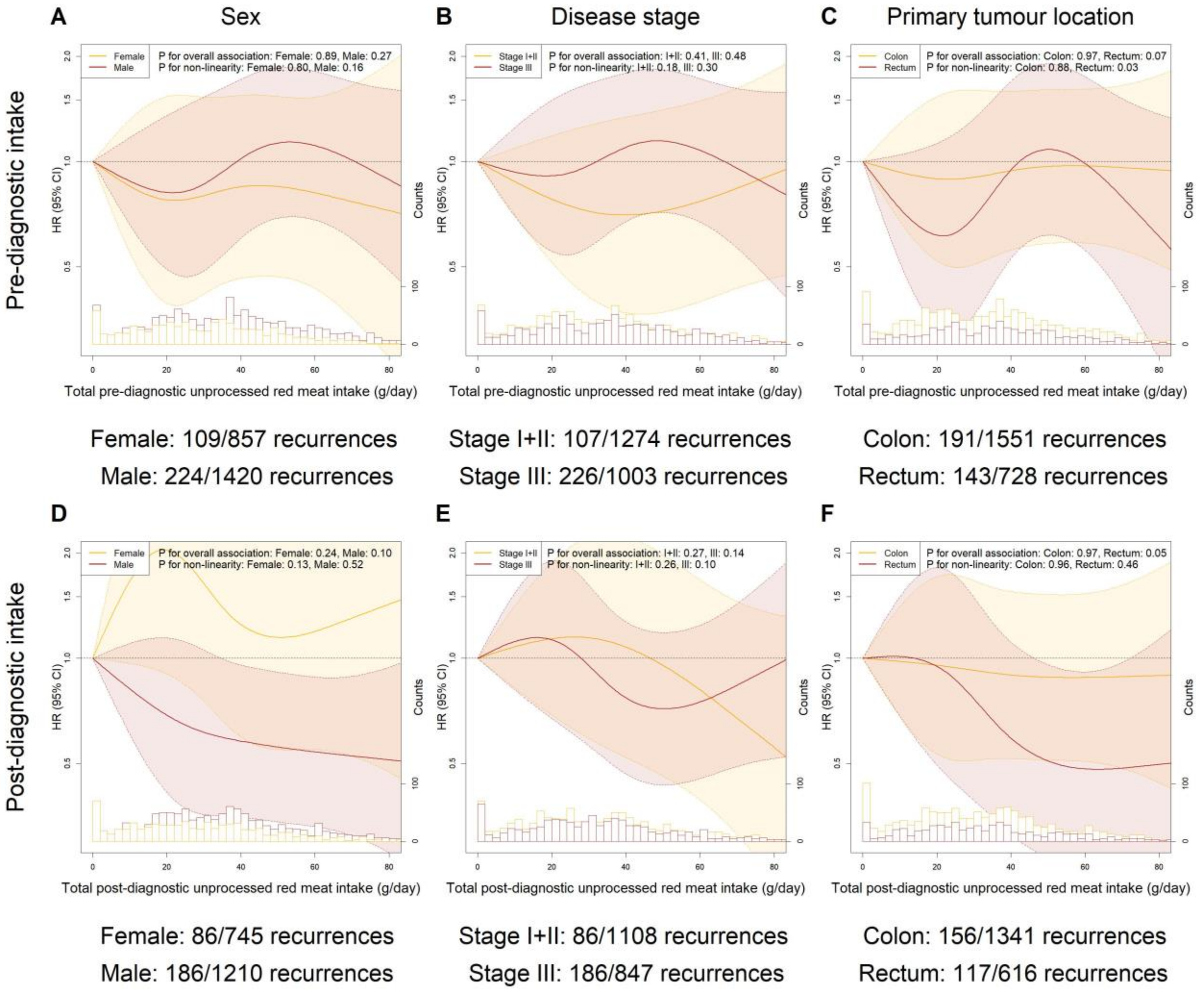

Fig S3 Pre- and post-diagnostic intakes of **unprocessed red meat** in relation to risk of **recurrence** stratified by sex (A, D), disease stage (B, E), and primary tumour location (C, F). The fully adjusted model included age, sex (except for analyses stratified by sex), education level, disease stage (except for analyses stratified by disease stage), primary tumour location (except for analyses stratified by primary tumour location), and total daily intakes of energy, low-fat dairy, and high-fat dairy. The numbers presented under each figure represent the number of recurrences in this subgroup and the total number of participants in this subgroup.

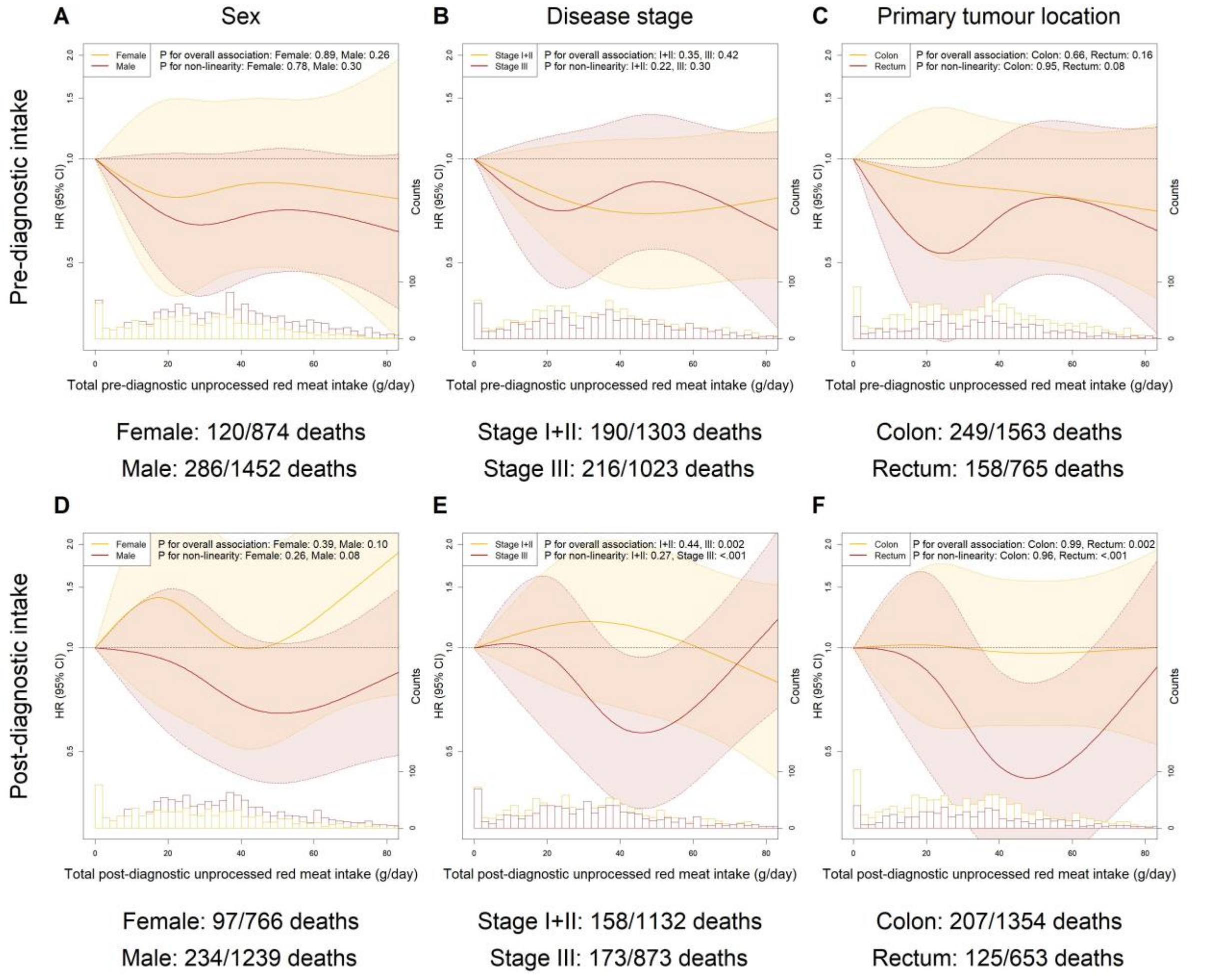

Fig S4 Pre- and post-diagnostic intakes of **unprocessed red meat** in relation to risk of **all-cause mortality** stratified by sex (A, D), disease stage (B, E), and primary tumour location (C, F). The fully adjusted model included age, sex (except for analyses stratified by sex), education level, disease stage (except for analyses stratified by disease stage), primary tumour location (except for analyses stratified by primary tumour location), and total daily intakes of energy, low-fat dairy, and high-fat dairy. The numbers presented under each figure represent the number of recurrences in this subgroup and the total number of participants in this subgroup.

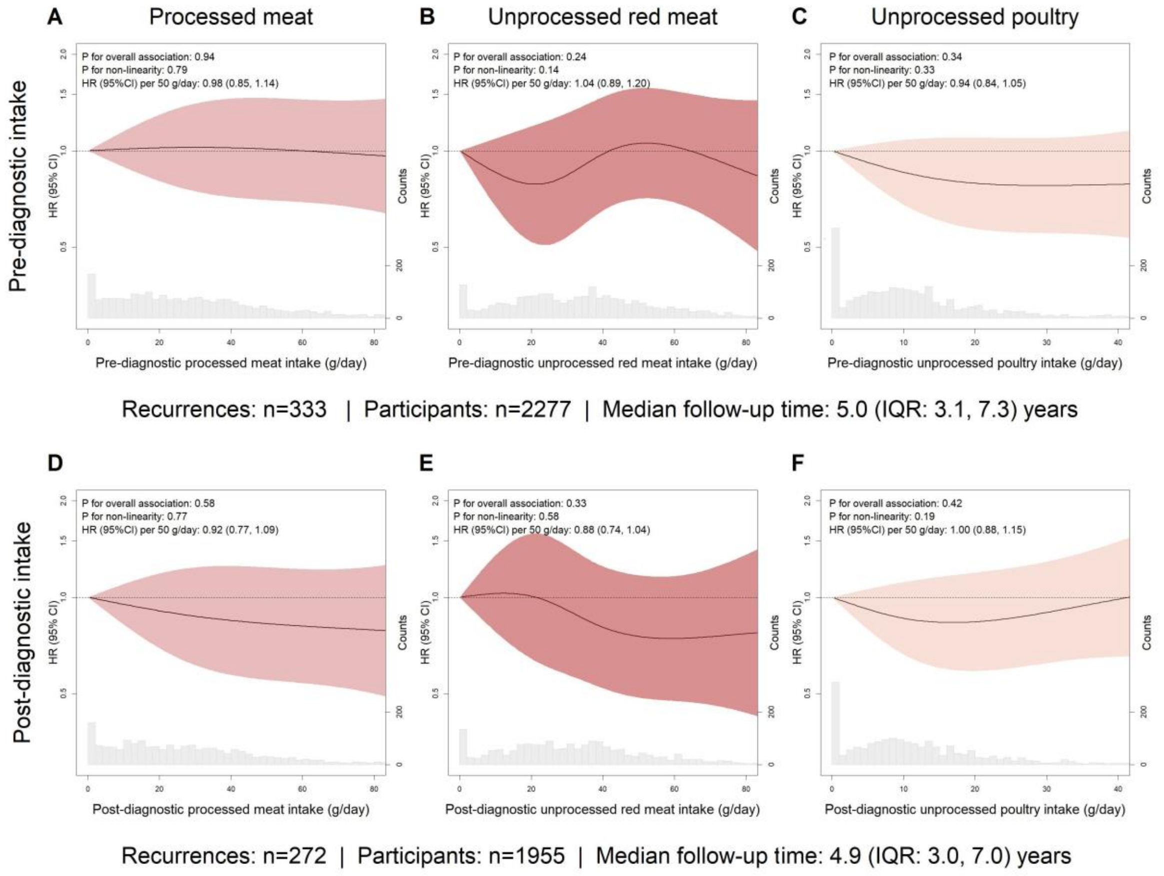

Fig S5 Pre- and post-diagnostic intakes of processed meat (A, D), unprocessed red meat (B, E), and unprocessed poultry (C, F) in relation to risk of **recurrence**, **when mutually adjusted for other meat types.** The fully adjusted model included age, sex, education level, disease stage, primary tumour location, and total daily intakes of energy, low-fat dairy, high-fat dairy, processed meat (except for analyses with processed meat as exposure), unprocessed red meat (except for analyses with unprocessed red meat as exposure), and unprocessed poultry (except for analyses with unprocessed poultry as exposure)

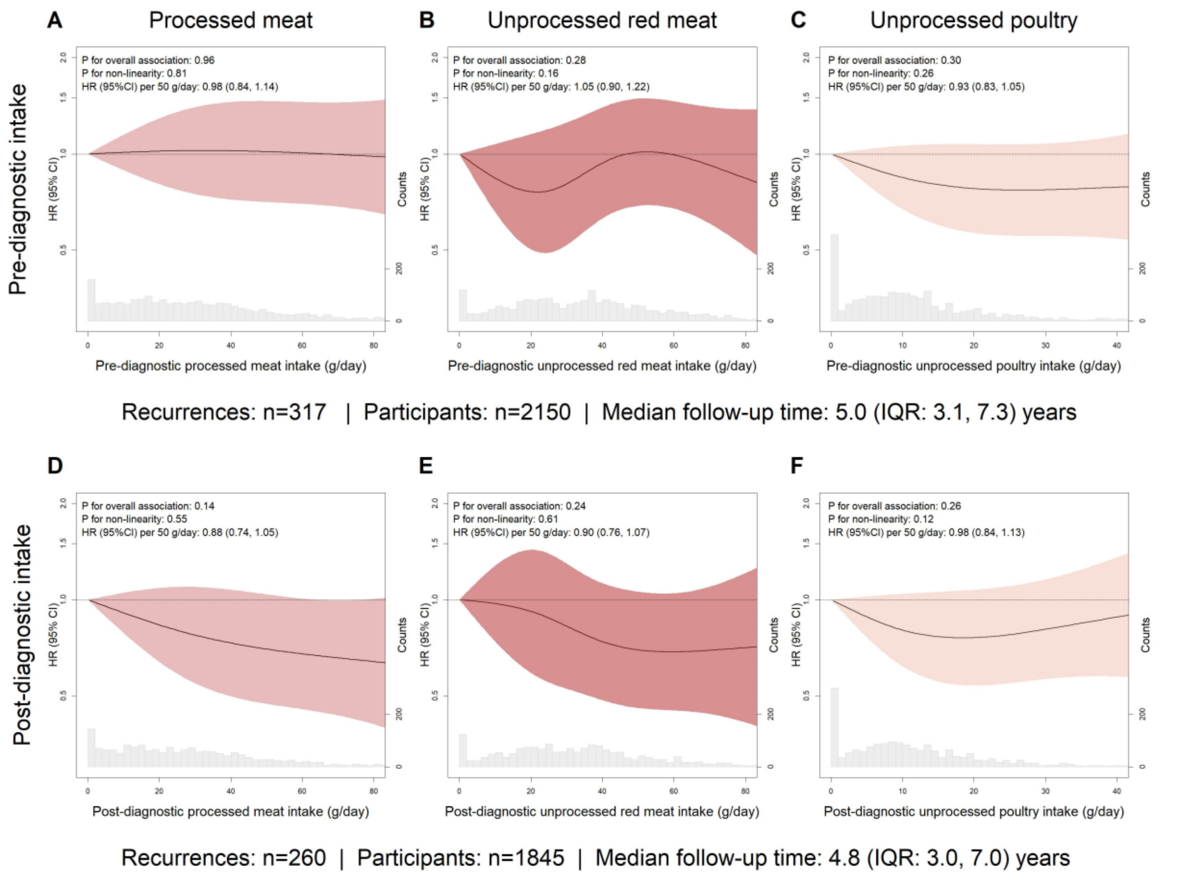

Fig S6 Pre- and post-diagnostic intakes of processed meat (A, D), unprocessed red meat (B, E), and unprocessed poultry (C, F) in relation to risk of **recurrence**, **when excluding participants who were younger than 50 years old at their diagnosis (n=128).** The fully adjusted model included age, sex, education level, disease stage, primary tumour location, and total daily intakes of energy, low-fat dairy, and high-fat dairy

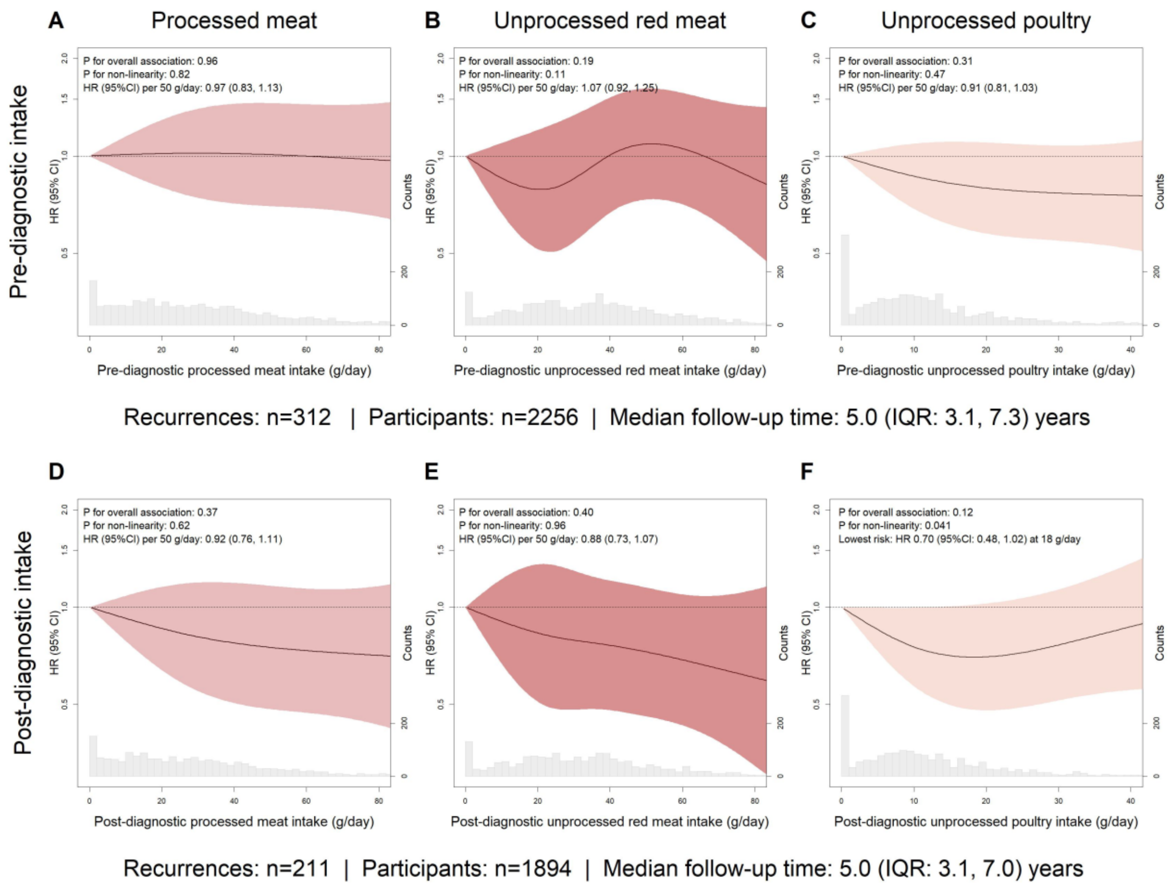

Fig S7 Pre- and post-diagnostic intakes of processed meat (A, D), unprocessed red meat (B, E), and unprocessed poultry (C, F) in relation to risk of **recurrence**, **when excluding those with a recurrence within 6 months after surgery (n=21) or within 6 months after the post-diagnostic FFQ (n=62).** The fully adjusted model included age, sex, education level, disease stage, primary tumour location, and total daily intakes of energy, low-fat dairy, and high-fat dairy

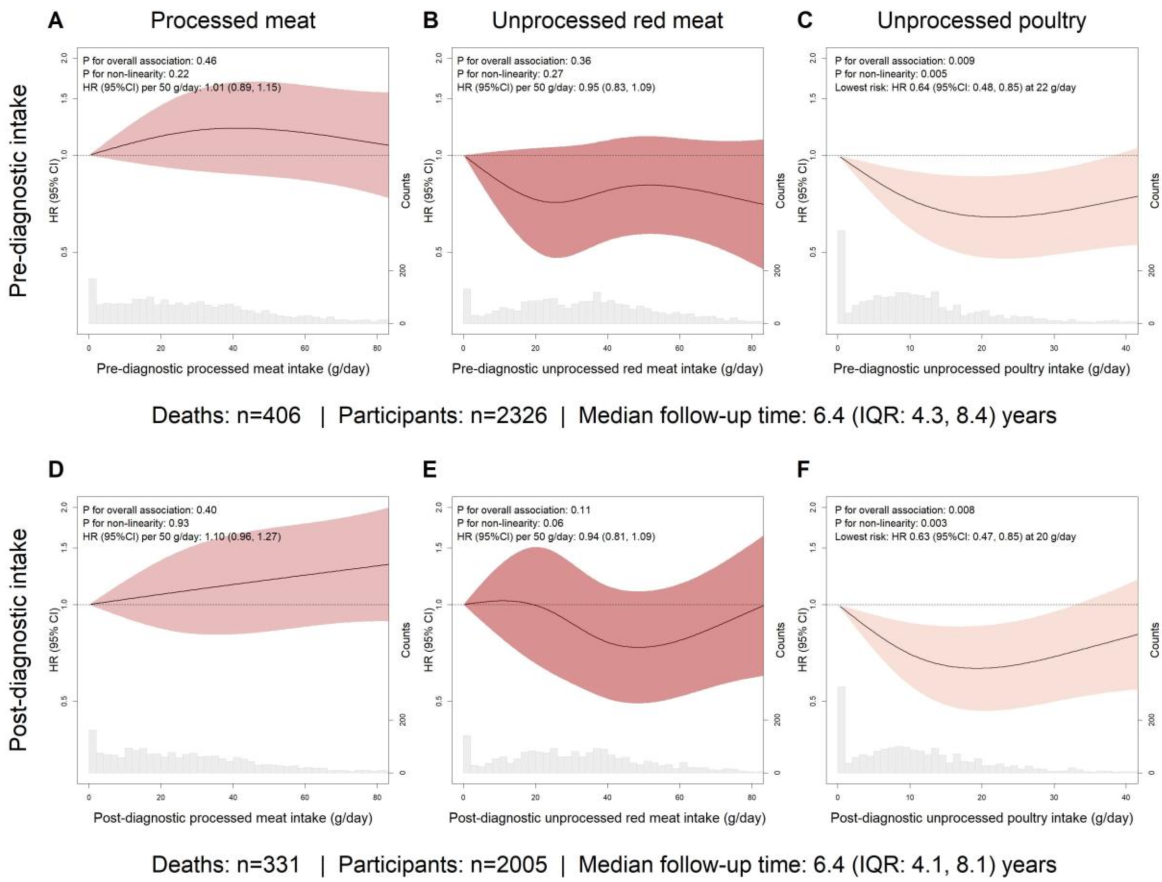

Fig S8 Pre- and post-diagnostic intakes of processed meat (A, D), unprocessed red meat (B, E), and unprocessed poultry (C, F) in relation to risk of **all-cause mortality**, **when mutually adjusted for other meat types.** The fully adjusted model included age, sex, education level, disease stage, primary tumour location, and total daily intakes of energy, low-fat dairy, high-fat dairy, processed meat (except for analyses with processed meat as exposure), unprocessed red meat (except for analyses with unprocessed red meat as exposure), and unprocessed poultry (except for analyses with unprocessed poultry as exposure)

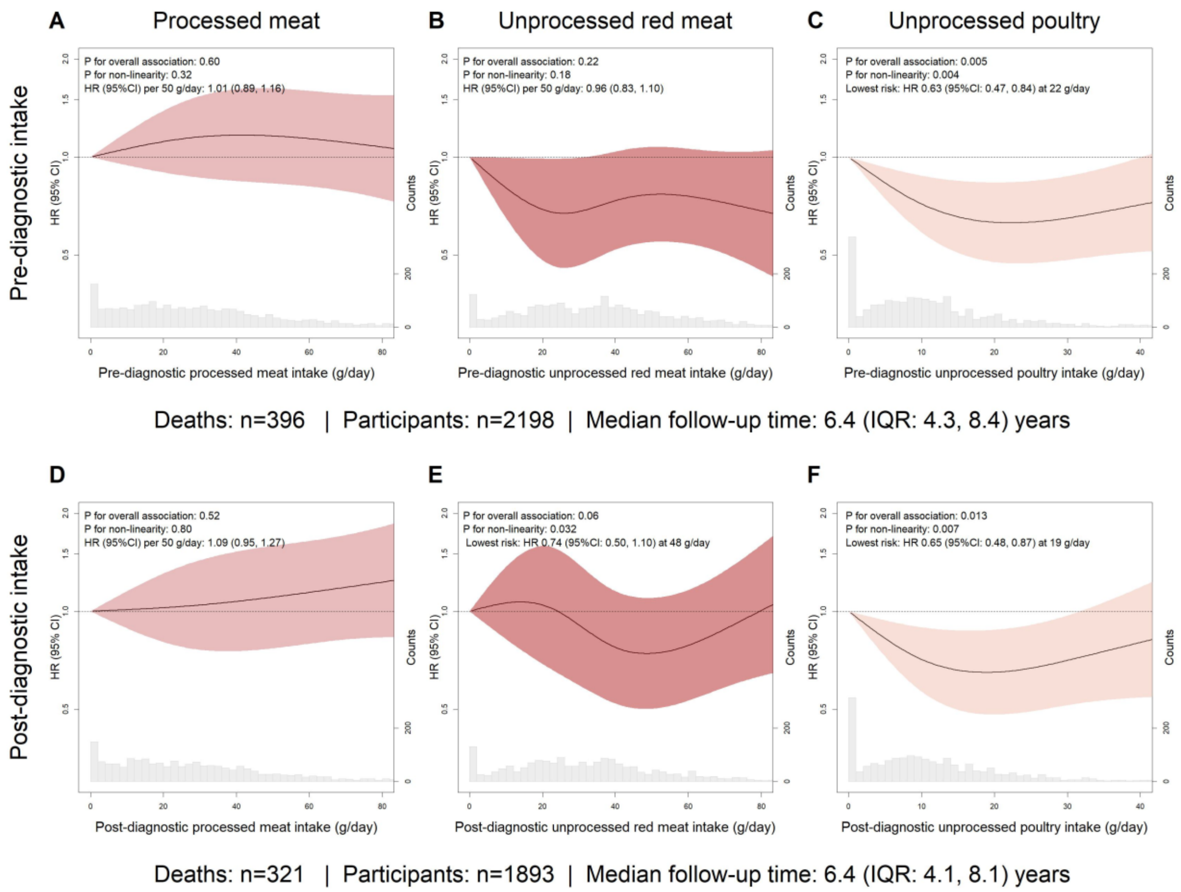

Fig S9 Pre- and post-diagnostic intakes of processed meat (A, D), unprocessed red meat (B, E), and unprocessed poultry (C, F) in relation to risk of **all-cause mortality**, **when excluding participants who were younger than 50 years old at their diagnosis (n=128).** The fully adjusted model included age, sex, education level, disease stage, primary tumour location, and total daily intakes of energy, low-fat dairy, and high-fat dairy

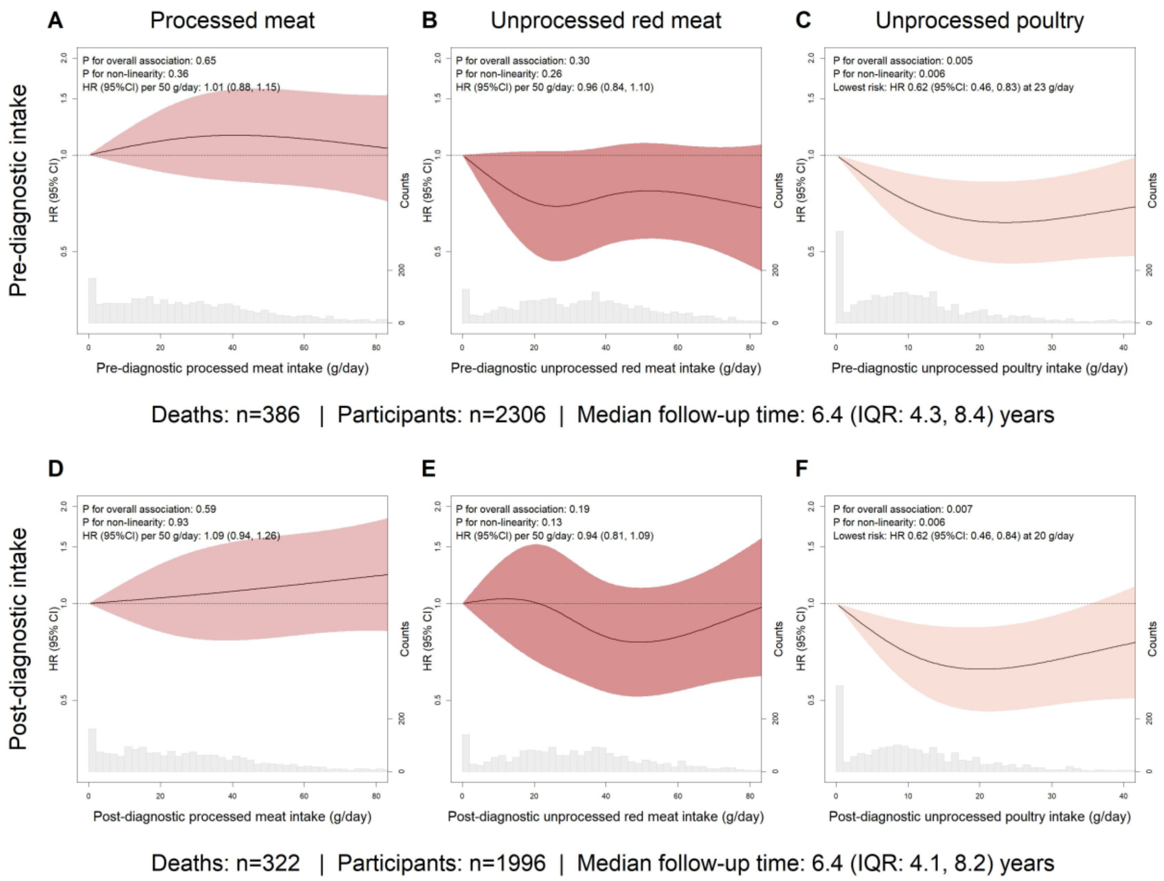

Fig S10 Pre- and post-diagnostic intakes of processed meat (A, D), unprocessed red meat (B, E), and unprocessed poultry (C, F) in relation to risk of **all-cause mortality, when excluding those who died within 6 months after surgery (n=20) or within 6 months after the post-diagnostic FFQ (n=9).** The fully adjusted model included age, sex, education level, disease stage, primary tumour location, and total daily intakes of energy, low-fat dairy, and high-fat dairy
